# Virtual immersive sensorimotor training (VIST) in collegiate soccer athletes: A quasi-experimental study

**DOI:** 10.1016/j.heliyon.2020.e04527

**Published:** 2020-07-24

**Authors:** Jennifer C. Reneker, W. Cody Pannell, Ryan M. Babl, Yunxi Zhang, Seth T. Lirette, Felix Adah, Matthew R. Reneker

**Affiliations:** a Department of Population Health Sciences, School of Population Health, University of Mississippi Medical Center, USA; b Department of Physical Therapy, School of Health Related Professions, University of Mississippi Medical Center, USA; c Department of Data Science, School of Population Health, University of Mississippi Medical Center, USA; d Athletic Department, Mississippi College, USA

**Keywords:** Neuroscience, Exercise, Musculoskeletal system, Neurology, Clinical research, Virtual reality, Sensorimotor control, Sports injury prevention

## Abstract

A burgeoning area of innovation in sports is the use of extended realities to provide athletes with novel training environments. Evidence has demonstrated that virtual environments can be useful therapeutic tools with demonstrated positive outcomes. The purpose of this pilot investigation was to determine the effects of virtual immersive sensorimotor training intervention by quantifying 1) the training effect measured via change in performance pre-to post-intervention on the virtual reality exercises, 2) the difference in the in clinical measures of functional sensorimotor control, 3) the injury incidence rate, and 4) on-field performance during soccer competitions. Statistical analyses were used to describe differences between an experimental and a control group. Participants were recruited from the men and women's soccer teams at two universities in the United States. Participants at one university were in the experimental group (n = 78) and received virtual immersive sensorimotor training, consisting of nine novel exercises in headset virtual reality, twice each week for six weeks. Participants at the second university were in the control group (n = 52). The virtual exercises were developed with reference to the rehabilitative principles of neuroplasticity to train various neurologic processes, contributing to overall sensorimotor control. This includes vestibular, visual and oculomotor activities, cervical neuromotor control training, movement coordination, and postural/balance exercises. The results indicate significant positive training effects pre-to post-intervention in seven of the nine training exercises (p ≤ 0.005) and improvement in clinical tests of cervical neuromotor control, balance, and inspection time (p ≤ 0.009) in the experimental group compared to the control. One of the virtual training exercises was positively associated with on-field performance (p = 0.022). No differences in injury rate or overall on-field performance metrics between the experimental and control were detected. This research study provides evidence of training and positive transfer from virtual to real-world environments, supporting the use of these novel virtual exercises to improve measures of sensorimotor control in healthy soccer athletes.

## 1 Introduction

Injury prevention in contact sports is of great importance for the health and safety of athletes. In the recent past, rule changes [1], equipment modification [2], and behavioral approaches have been implemented to decrease player risk for concussion and musculoskeletal injuries [3, 4]. In addition to these approaches, research has demonstrated that sensorimotor training (either at the granular or composite level) may also add a protective factor against sport injuries [5, 6]. This is intuitive, given that simultaneous processing of sensory information with coordinated precision of motor responses are required to produce the speed, balance, and agility necessary for success and safety on-field [7]. It has been shown that sensorimotor control exercises can be effectively delivered to groups of athletes [5, 8]. Conditioning the sensorimotor control system as a component of sports-team-training presents the potential to enhance a healthy, efficient neuromotor system [5].

A burgeoning area of innovation in sports is the use of three-dimensional extended realities (XR) to provide athletes with novel training environments [9, 10]. XR includes immersive virtual reality (IVR), where a user is only able to see and interact with a virtual environment, and augmented reality (AR), where the virtual environment is overlaid upon the real-world environment, and the user can see and interact with both. While originally developed primarily for gaming, many researchers in healthcare are finding ways to incorporate two and three-dimensional extended realities into their profession [11, 12, 13, 14]. These technologies enable manipulation of the user's context of and interaction with a sensory-stimulating environment and may provide an ideal training and rehabilitative interface for various components of sensorimotor control [15, 16, 17, 18, 19]. While offering new opportunities, potentially increasing motivation to engage in exercise, the way in which user's interact with and process the virtually derived sensory stimuli and how this impacts motoric responses is a subject of intense investigation [17, 20].

Evidence has demonstrated that virtual environments, whether provided through a head-mounted device, where the device encapsulates the user's eyes and displays the virtual environment through stereoscopic Fresnel lenses, or in an immersive whole-room system, which utilizes projectors in an instrumented room to produce an artificial environment, can be useful therapeutic tools for assessment or treatment, with promising positive outcomes [12, 15, 16, 18]. Typically, studies exploring IVR have been described in individuals with known functional limitations and impairments. Studies exploring the usefulness of IVR to train healthy athletes as a component of a strength and conditioning training program have not been described. One small study included the use of IVR as a component of a multi-modal training regimen in athletes [5]. Although the results from this initial study were promising, it is unknown whether a training intervention, delivered primarily in IVR, to healthy collegiate athletes is effective at enhancing sensorimotor/neuromotor control.

Therefore, the purpose of this investigation was to determine the results of a sensorimotor training intervention delivered using IVR to an experimental group compared to a control group by quantifying 1) the training effect measured via change in performance pre-to post-intervention on the IVR exercises, 2) the difference in the in clinical measures of functional sensorimotor control, 3) the injury incidence rate, and 4) on-field performance during soccer competitions. The hypothesis was that there would be training effects on the IVR exercises and improvements in clinical measures of sensorimotor control. Secondarily, we hypothesized that there would be a positive association between IVR score and on-field performance and an inverse relationship between IVR score and injury risk.

## 2 Materials & methods

Trial design: This study was conducted as a pilot, quasi-experimental design with two participating institutions. This research protocol was registered prospectively on clinicaltrials.gov (NCT04036916) and there were no changes to the methods after trial commencement. The study was approved by the University of Mississippi Medical Center Institutional Review Board (IRB), the University of West Alabama and Mississippi College IRBs prior to initiation. Informed written consent was received prospectively and the rights of each participant was protected.

Participants: Participants for the study were recruited from the men's and women's soccer teams at Mississippi College (MC) in Clinton, MS, USA (selected to be the experimental group) and the University of West Alabama (UWA) in Livingston, AL, USA (selected to be the control group). Both institutions are Division II members of the Gulf South Coast Conference and the National Collegiate Athletic Association (NCAA). Study recruitment was completed when the athletes reported to campus for the 2019 season. All soccer athletes eligible to play in the 2019 season, aged 18 or over, were invited to participate. Exclusion criteria were a current diagnosis of concussion (i.e. non-medically cleared), lower-extremity musculoskeletal injury, seizure disorder with photosensitivity, or any other diagnosis that would prevent the athlete from participating. A total of 130 participants were included (mean age = 20.5; standard deviation age 1.6; 56% male).

The Virtual Environment: The Oculus Quest was used as the IVR device to deliver the intervention. The Quest was released in May 2019 and is considered a high-fidelity, stand-alone device (i.e. no computer tether required) with inside-out, six degrees-of-freedom headset tracking and hand tracking through two manual touch controllers. The Quest utilizes dual adjustable lenses, has a refresh rate (frame rate) of 72 Hz, and the display resolution is 1440 X 1600 per eye with a horizontal field of view of approximately 90°.

Within the intervention, participants completed nine novel training exercises in headset IVR. The training exercises were intended to augment the user's sensorimotor control through the use of novel, visually stimulating virtual environments, to produce desired motor responses while the user's vestibular and proprioceptive systems in the real-world were active and accurately represented within the virtual world (i.e. a user's head rotation in the real world would produce the same rotational change in the virtual environment or postural sway in the real world would produce corresponding visual shift in virtual environment). Because of the potential for virtual experiences to create sensory-motor mismatches, often by producing the visual illusion of translational movement (i.e. flying or walking) with no corresponding physical movement [17], the exercises did not include virtual translational movements. Additionally, no third-person avatars or representations of the participant's physical body were produced in the virtual environment. Rather, all of these exercises were completed from the first-person perspective. A beam of light emitting from the hand controller or headset was represented within the virtual environment to select or trace the path of an object.

Experimental Training: The intervention delivered to the participants in the experimental group was six weeks long, consisting of two face-to-face sessions per week for each athlete. All sessions were conducted in the strength and conditioning facility at MC. All IVR exercises were completed while standing unless a participant sustained a musculoskeletal injury, making standing unsafe or unable to be tolerated. In this case, the athlete completed the exercises in sitting until cleared by their university athletic trainer. A description of the basic requirements for each of the training activities can be found in Table 1.

**Table 1:** VIST exercise descriptions.

Name of exercise	Description of requirement for level zero (most basic, training and testing level)
Smooth Pursuits	A small object was presented that moved on an invisible spline, in an unpredictable manner, across the visual field, near and far from the eyes. Participants were to follow the object with their eyes (head still) and concurrently trace the path with a collider on a hand controller. Points were achieved according to ability to keep the collider in contact with the object.
Saccades	A small object was presented which rapidly and unexpectedly moved, in an unpredictable manner, across the visual field, near and far from the eyes. With the head still, participants were required to use a saccadic eye movement to locate the new position of the object and select it with a manual pointer on a hand controller. Points were achieved according to the number of selections (hits).
Near Point Convergence	A small object was presented which moved close and far from the eyes in the virtual environment. Participants were required follow the path of the object with their eyes and use the manual controller to align a second object with the first object as it moved. Points were achieved according to time the second object was in contact with the first object.
Peripheral Vision	A small colored object was presented in the center of the screen while four colored blocks on the left and right side of the screen were also presented. All the objects changed color each time a selection was made. Participants were to look at the central object, use their peripheral vision to identify the block with the same color, and select that block with the manual pointer on the hand controller. Points were achieved according to the number of correct selections (hits).
Visual Figure	A specific object was hidden in a visually complex environment. Participants utilized visual scanning to locate the object and then select it with a manual pointer on a hand controller. Each selection resulted in a shuffling of the environment and a new location of the hidden object. Points were achieved for each selection of the hidden object within the timeframe.
Joint Position Target	A bulls-eye target was presented centrally in the visual field. A pointer from the center of the headset enabled the participant to align their head with the center of the target. The participant was instructed to look right, left, or up to cause the screen to go black. Then the participant was instructed to return their head to the beginning location (using cervical proprioception without visual input). Once returned to midline, a manual trigger was depressed, revealing how close to the original location they were. Points were achieved based upon overall error in repositioning from center. Three repetitions were completed in each direction (this was not timed).
Neuromotor Maze	A complex map was presented in the IVR headset. A small object, which was controlled by the position of the participant's head, was presented. The participants were required to navigate the object through the map as accurately and fast as possible (using cervical neuromotor control). Errors off the map produced a negative sound. Points were accumulated as they made it through the map on the ascribed path (speed and accuracy).
Cervical Posture	The exercise required participants to move between a neutral head position and a proper cervical retraction with a 3 s hold. Points were accumulated by achieving correct cranial-cervical flexion and return to neutral.
Balance	A visually tilting environment was presented to the participants, which slowly changed between a left and right horizontal tilt. Participants were required to maintain postural stability in standing, minimizing sway. Score was based upon the amount of postural sway.

The intervention was named VIST (Virtual Immersive Sensorimotor Training). The VIST intervention exercises were developed to require multi-sensory integration (including visual, vestibular, and proprioceptive inputs) and a skilled motor output to achieve an action goal. This included oculomotor activities (smooth pursuit, saccade, and binocular convergence), cervical neuromotor control exercises, coordination (i.e. multi-segment complex movements), and postural/balance exercises. Each training exercise was established with three levels to incrementally progress in difficulty and complexity across time. Difficulty was progressed by changing multiple variables, including, 1) the parameters required for success with the action goal, 2) the type and number of sensory inputs to be integrated, and 3) the coordination of movement (e.g. simultaneous control of head/neck and oculomotor movement to achieve the action goal). The environment in the IVR headset was also manipulated to increase the complexity of the visual input during task completion and various standing postures (comfortable, narrow, tandem) and a compliant surface (2.25″ thick, closed-cell foam pad) were used to add a balance challenge. Three or four of the VIST training exercises, difficulty level, and standing position on or off foam were chosen in advance for each training session by the study team. To keep the training interesting for the participants and to train the various components of each subsystem equitably, the exercises selected for the sessions (grossly) represented at least one primarily oculomotor activity, one visual perceptual activity, and one cervical training activity. At the sessions, each training exercise lasted 60–90 s in duration and repeated in series for 15 min. The completion of each exercise at each session resulted in a score, which was presented to the user in IVR and represented a metric of their success with completion of the exercise (for mastery-oriented individuals). In addition, a virtual leaderboard was used to add an element of competition between the participants (for performance-oriented individuals) [21]. This was done to increase motivation, improve effort and overall performance during training.

The control group was treated as a true control and completed no VIST intervention.

Athletic Environment: Each of the four participating teams had an independent coaching staff who autonomously made decisions regarding game, practice, and training schedules and activities. Each team also had a university assigned athletic training staff member who conducted evaluation and care of athlete injuries per their standard protocol. The research team did not influence or direct any of these activities. After the soccer teams at MC participated in a research project in the previous year which included the Iron-Neck [5], the strength and conditioning coaches opted to use this device as their preferred method for neck strengthening in the soccer athletes. The Iron-Neck exercises were completely overseen by the strength and conditioning coaches. It is included here because of the strong likelihood in contributing to some of the outcome metrics of this project. Conversely, as per the standard of practice at UWA, soccer athletes did not participate in any formal neck strengthening with the Iron-Neck during the soccer season.

**Figure 1:**
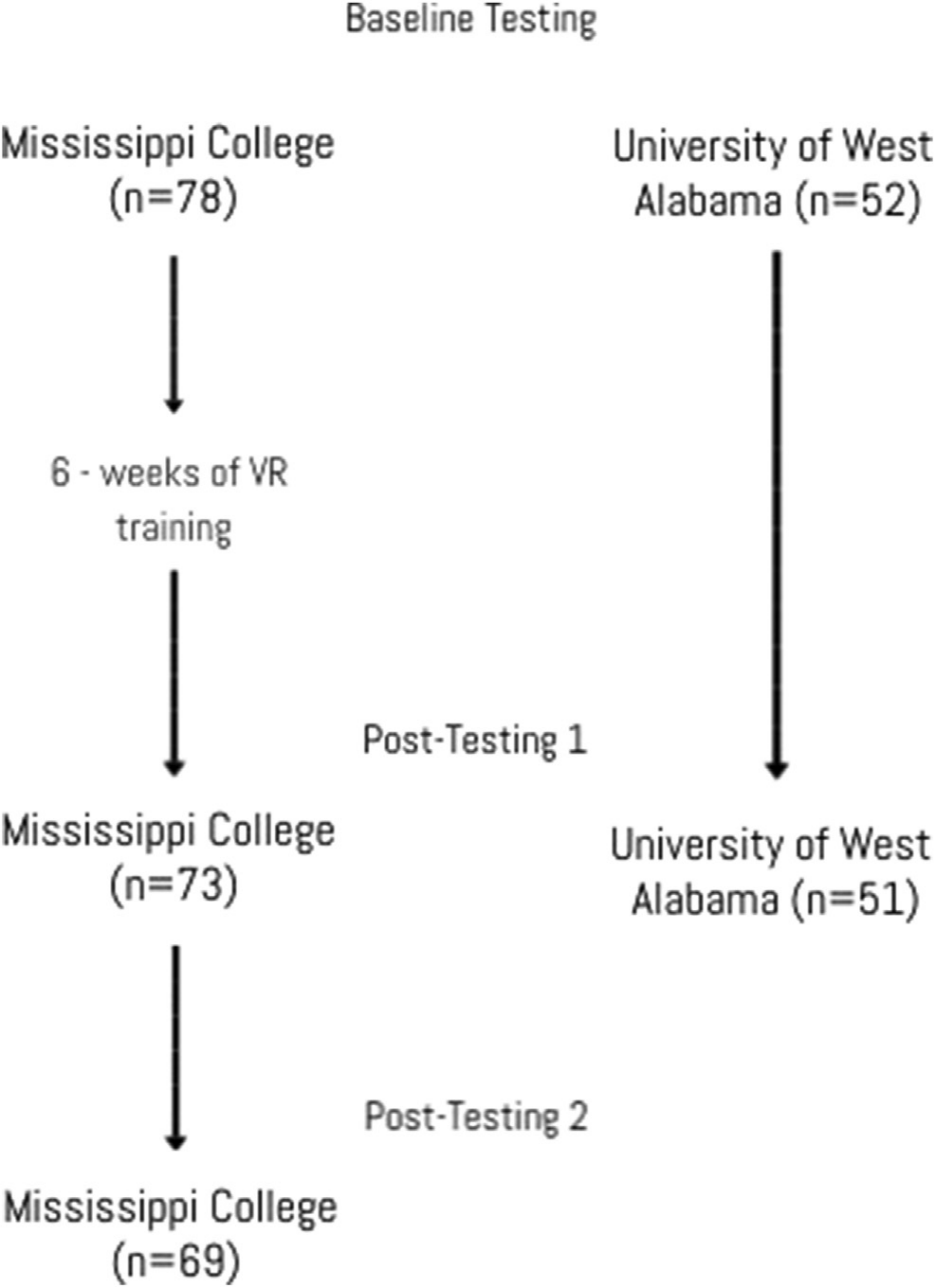
Flow diagram.

Outcomes: The primary outcomes were measured pre- and post-participation in the six-week intervention (or after six weeks had passed since baseline for the control). A third measurement (follow-up) was also completed at the conclusion of the soccer season for the athletes at MC to determine if there were changes in the training effect or clinical test performance after training was ceased. The follow-up testing was completed 10 weeks after the training intervention concluded (Figure 1).

*Training effect:* The first main outcome is the training effect from the IVR exercises themselves. To measure training effects, each IVR exercise had a testing level, which lasted for one minute (with exception of Joint Position Target) and produced the exact same virtual environment each time it was done. Performance on these activities resulted in accumulation of points and a total score in like-fashion to playing a game. The lowest score was zero, but the upper possible limit was not defined.

*Near-Effects:* These are considered closely related sensorimotor and neuromotor control abilities which have potential for improvement in response to the VIST intervention. The near-effects were quantified through commonly used clinical tests and measurements, described in Table 2. These outcomes were chosen because they have been used in research to measure various domains of sensorimotor and neuromotor control and have demonstrated clinical usefulness and psychomotor soundness [5, 22, 23, 24, 25, 26, 27, 28, 29, 30, 31, 32].

**Table 2:** Clinical outcome measures.

Outcome	Description of method of measurement
Smooth pursuit and saccade eye tracking	Eye tracking was completed using the Tobii Eye Tracker 4C. With a frame rate of 90 Hz, this eye tracker was mounted to a 15″ laptop computer. Participants were seated with their feet on the ground approximately 23″ from the computer screen. For smooth pursuit, participants followed a sine wave at .1 Hz, .2 Hz, .3 Hz and .4 Hz. For saccades, users performed non-anticipatory pro-saccadic movement left, right, up and down (3X in each direction). Eye tracking was recorded according to the pixel location for each eye in reference to the object location on the screen. Spike 2 and MATLAB software was used to process all the data to identify slope, gain, and phase (in degrees) measurements for smooth pursuits and latency (in milliseconds), peak velocity (in degrees per second), duration (in milliseconds) and amplitude (in degrees) for saccade parameters.
Near Point Convergence (NPC)	NPC was tested by asking each participant to hold a 21″ convergence ruler (Bernell, Vision Training Products, Inc.) against the tip of their nose and slowly move a slide card towards their face. The slide card was marked with an 11-point font “X”. The participant was instructed to stop the slide-card along the ruler where diplopia of the “X” was reported. The test was completed three times. The mean distance of NPC was calculated and recorded in centimeters [25].
Static Balance and Reaction Time measured by the Sway Balance App	The Sway Balance App (SwayMedical.com) is a medical application intended to be downloaded and used on an appropriate smartphone or similar device and is supported in research literature as a valid and reliable baseline assessment of static balance in athletes. Participants completed this test by using their personal device to download and access the Sway App. Sway senses and records movement using the triaxial accelerometer located in the smart device. A proprietary algorithm is used to calculate postural sway and reaction time on a 0–100 point scale, where 100 is perfect. Balance: The balance score, includes measurement of postural sway in five standing positions for 10 s, all with the eyes closed. Total score is calculated across trials in each of the five standing positions. One practice test and two trials were completed for each stance. Score was calculated from the two trials. Reaction Time: Simple reaction time was based on movement of the phone in response to a screen color change. Impulse control was based upon movement of the device with the appearance of a green colored screen, and refraining from movement with the appearance of a red colored screen. Inspection time is a complex measurement of reaction time. This test requires the participant to determine which line, presented on the left or right side of the screen is longer. One practice test and two trials were completed for each type of reaction time. Each type of reaction time was completed five times per trial. The fastest and slowest reaction time was dropped and trial score was recorded as the mean across the remaining three attempts for each type of reaction.
Deep Neck-Flexor Endurance Test (DNFT)	The participant was positioned in supine crook-lying on a mat table with hands on abdomen. The tester instructed the participant to maximally “tuck the chin” and lift the head 1” (2.5 cm) and hold. The tester began timing at the initiation of the lift. During testing, the examiner slid 2 stacked fingers under the participant's head to provide tactile cue for maintaining the listed position. Time was ended when the participant met 1 of 4 criteria: losing the chin-tuck; resting the head on the examiner's fingers for >1 s; raising the head above 1”; or becoming unwilling to continue. One correction by the examiner of a deviation in position was permitted through verbal cue [29].
Cranial Cervical Flexion Test (CCFT3)	The participant was positioned in supine crook-lying on a mat table with hands on abdomen and asked to recruit the deep neck flexors in a precise manner according to the description of Stage 1, as described by Jull [27]. This test was used to measure the ability to recruit and control the deep cervical flexors. The highest level, as measured by the Stabilizer biofeedback device (Chattanooga), 22, 24, 26, 28 or 30 mmHg, which the participant could achieve and hold for 3 s with the correct muscle action, without observation or palpable contraction of the superficial flexors was recorded. One trial of this test was completed.
Cervical Isometric Strength	The Activ5 (ActivBody.com) was used to measure cervical muscle strength in each direction (flexion, extension, rotation, and side-bending). This device connects to a smart device through an App and measures force production in real-time. Participants were seated in a chair, with their back unsupported, feet on the ground, and head in a neutral position throughout testing. All testing was completed with the participant holding the device against their head according to instructions and oversight provided by a licensed physical therapist. A practice test and two trials were completed. Force was calculated in two ways: based on percentage of total body weight and raw force in pounds. Each outcome was calculated as the mean of the two trials.

*Far-effects:* Ultimately, this training is being explored to determine if its impact can be detected for on-field outcomes, including game performance and injury risk. These are considered far-effects as they are not closely related to the VIST activities themselves and are heavily influenced by many other factors, including those internal and external to the control of an individual player. Detecting an association between one or more of the IVR training exercises and these far-effects is potentially informative on which sensorimotor abilities are more important in soccer. These measurements were accumulated across the soccer season.

*Injury incidence rate:* For both institutions, injury was defined according to the NCAA-Injury Surveillance program and included concussions and traumatic lower extremity musculoskeletal injuries [33]. This method has been described in previous research [5]. For our purposes, a reportable injury must have occurred during participation in a NCAA-sanctioned practice or competition, requiring attention from an athletic trainer, and resulting in missed athletic days. Contusions and overuse injuries were not included. The injury rate was based on athlete exposures (AE). Each AE was defined as one student-athlete participating in one practice or competition, with the possibility of injury. One AE was counted regardless of the time of participation and were reported in the Countable Athletic Related Activities (CARA) logs [33]. The participating universities collected injuries through the athletic trainer assigned to each team and tracked CARA activities as standard practice.

*On-field performance*: Each regular season competition was video-taped by the participating institution. These videos were analyzed on-line by InStat (InStat, Moscow, Russia), a commercial soccer performance evaluation company. To produce an InStat rating for an individual athlete, athletes had to participate for a required minimum amount of time in a competition and complete a certain number of on-field actions. Athletes were evaluated on a variety of performance outcomes, specified according to their position on the team, including those measuring accuracy, ball challenges, shots-on-goal, tackles, interceptions, ball recoveries and lost balls. While considering the athlete's position on the team, the level of play, and opponent skill, a proprietary algorithm was used to derive one overall performance measure, called the InStat Index. A higher index score is indicative of better performance. Many collegiate and professional teams utilize InStat to measure game performance and it has been used as a game-performance measurement in related research [34].

Statistical Methods: Basic characteristics were reported for demographic and injury history of the groups at baseline. Regression models with 1-sided tests were used to investigate whether the training has a positive effect on score changes from baseline to post-tests for the IVR activities and the clinical tests between the experimental and control participants. Individual baseline test scores and concussion history were controlled in the regression model. Ordinal logistic regression was conducted for the CCFT test. To compare change in outcomes in the experimental group between post-test and follow-up, 2-sided paired t-tests and Wilcoxon signed-rank test were completed. To further evaluate if the training that MC athletes received during the six-week intervention carried over with effects at follow-up, linear regression and ordinal logistic regression models were used to test whether score changes during the period of non-training (i.e. from post-test to follow-up of MC students and from baseline to post-test of UWA) were similar, controlling for concussion history and performance scores at the previous testing session (i.e. baseline for UWA and post-test for MC). Any athlete who sustained a concussion between baseline and post-test or post-test to follow-up were removed from the corresponding analysis.

For the eye tracking data, Spike 2 and MATLAB software were used to filter the data and quantify the relevant eye movement parameters. The smooth pursuit tests were examined by the four sine wave frequencies, 0.1–0.4 HZ. The gain and phase were used to examine the amplitude and the latency of eye tracking in the vertical direction. A gain equal to 1.0 indicates an ideal smooth pursuit, but a gain of between 0.85 and 1.0 was considered within the normal range. The slope was used to examine the amplitude of the eye tracking in the horizontal direction. Eye movement parameters for saccades by four directions were examined, including latency, peak velocity, duration, and amplitude.

Injury rates per 1000 exposures were calculated by mixed effects Poisson models with random intercepts for the individual players and offsets based on individual injury exposures. Expected marginal injury rates were estimated and multiplied by 1000. Similarly, mixed effect Poisson models with random intercepts and offsets were used to model incidence rate ratios for total injuries as predicted by IVR scores at baseline and post-intervention. This was accomplished via a triple interaction term between respective IVR exercise, a pre/post intervention indicator, and an indicator for whether the athlete was the experimental or control group. The appropriate estimates were then extracted from linear combinations. All models were additionally adjusted for sex and previous concussions. InStat Index scores, as a function of VIST exercise scores, were estimated from random intercept-slope models adjusting for sex and whether the player was a member of the experimental or control group. All analyses were completed in R (The R Project for Statistical Computing), SAS (SAS Institute, Inc.), and Stata (StataCorp).

## 3 Results

Baseline demographic characteristics for the participants are presented in Table 3. There were 78 athletes enrolled from MC, and 52 from UWA. Approximately 44% of the participants at each school were female and a majority of the sample were white (84.6% at MC and 68.6% at UWA). At MC, 28.2% reported a history of previous concussion, 42.3% at UWA. Of the participants, 64.1% at MC and 61.5% at UWA acknowledged a history of prior lower extremity injury. The mean age of the participants was 20 years.

**Table 3:** Baseline demographic characteristics.

	MC (n = 78)	UWA (n = 52)
Female, n (%)	34 (43.6)	23 (44.2)
Race: White, n (%)	66 (84.6)	35 (68.6)
Race: Black or African American, n (%)	6 (7.7)	8 (15.7)
Race: Asian, n (%)	0 (0)	3 (5.9)
Race: Hispanic or Latino, n (%)	6 (7.7)	5 (9.8)
Concussion History, n (%)	22 (28.2)	22 (42.3)
Leg Injury History, n (%)	50 (64.1)	32 (61.5)
Age, y	20.01 (1.6)	20.12 (1.6)

Regression results for the training activities and functional performance measures are presented in Table 4. The table presents the training effects, as measured by the IVR activities and the near-effects, as measured by the traditional clinical tests. The coefficient estimates provide the mean improvement in score between baseline and post-intervention testing for the individuals in the experimental group as compared to individuals in the control group, and the 97.5% confidence interval lower bounds are given to show the range of plausible improvement. Group means and standard deviations at baseline and post-test are available in Table 5.

**Table 4:** Regression analysis of score changes from baseline to post-test evaluating training effects.

Tests	Coefficient Estimates (Std Err)	97.5% CI Lower Bound	P-value
*IVR Exercise Scores*			
Smooth Pursuits	74.18 (14.5)	45.44	<.0001
Saccades	95.78 (13.6)	68.92	<.0001
Near Point Convergence	38.02 (14.7)	8.99	0.005
Peripheral Vision	43.98 (8.6)	27.01	<.0001
Visual Figure	127.73 (12.6)	102.74	<.0001
Joint Position Target	41.93 (11.1)	20	0.0001
Neuromotor Maze	41.96 (10.7)	20.66	0.0001
Cervical Posture	11.22 (19.1)	-20.62	0.279
Balance	1.83 (19)	-35.74	0.462
*Clinical Test Scores*			
Near-Point Convergence	-0.33 (0.2)	-0.79	0.923
CCFT Highest 3 Second Hold	0.89 (0.2)	0.502	<.0001
DNFT Time Held (Seconds)	8.63 (3.6)	1.55	0.009
*Cervical Isometric Measurements*			
Flexion (Force)	11.38 (2.4)	6.55	<.0001
Flexion (Percent)	6.82 (1.4)	4.01	<.0001
Extension (Force)	8.23 (1.9)	4.47	<.0001
Extension (Percent)	4.84 (1.1)	2.64	<.0001
Left Side Flexion (Force)	6.15 (1.8)	2.61	0.0004
Left Side Flexion (Percent)	3.71 (1.1)	1.51	0.001
Right Side Flexion (Force)	8.18 (1.7)	4.85	<.0001
Right Side Flexion (Percent)	5.01 (1.1)	2.91	<.0001
Left Rotation (Force)	5.97 (1.5)	3.04	<.0001
Left Rotation Flexion (Percent)	3.56 (1)	1.54	0.0004
Right Rotation (Force)	6.21 (1.5)	3.32	<.0001
Right Rotation Flexion (Percent)	3.92 (1)	2	<.0001
*Sway Scores – Balance*			
Feet Together	6.89 (2.2)	2.55	0.001
Tandem Right	7.18 (2.6)	2.04	0.003
Tandem Left	4.65 (1.8)	1.1	0.005
Single Leg Stance (R)	8.66 (3.1)	2.47	0.003
Single Leg Stance (L)	9.72 (3)	3.87	0.0007
Overall Balance	6.04 (1.6)	2.93	0.0001
*Sway Scores – Reaction Time*			
Simple	0.65 (1.4)	-2.07	0.319
Impulse Control	0.73 (1.1)	-1.48	0.257
Inspection	1.9 (0.8)	0.36	0.008

IVR = Virtual Reality; CCFT = Cranial-Cervical Flexion Test; DNFT = Deep-Neck Flexor Test: all cervical isometrics measured in pounds of force; percentage should be interpreted as percent of max force calculated based off total body weight. All balance stances were completed with the participants eyes closed.

**Table 5:** Outcome characteristics, Mean (SD), of baseline to post-test scores.

	MC		UWA	
	Baseline	Post-test	Baseline	Post-test
*VR Exercise Scores*				
Smooth Pursuits	132.23 (66)	234.45 (92.1)	131.41 (68.1)	159.67 (78.4)
Saccades	300.94 (108.6)	427.03 (68.1)	283.94 (101.1)	328.92 (87.6)
Near Point Convergence	84.8 (59.5)	184.08 (98)	89.39 (74.8)	152.06 (94.7)
Peripheral Vision	274.69 (66.7)	342.14 (47.4)	277.31 (50.3)	297.57 (48)
Visual Figure	277.24 (75)	409.93 (74.2)	274.52 (62.1)	284.31 (77.3)
Joint Position Target	182.9 (67.5)	237.63 (49.8)	151.55 (72.4)	179.35 (77.4)
Neuromotor Maze	227.27 (69.6)	292.22 (72.1)	201.04 (56.2)	232.39 (55.3)
Cervical Posture	330.45 (88.4)	314.63 (101.3)	312.06 (88.3)	304.69 (99.2)
Balance	230.46 (93.1)	253.07 (112.1)	242.82 (87.8)	261.02 (112.1)
*Clinical Test Outcomes*				
Convergence	2.16 (2.3)	2.02 (2)	1.8 (1.7)	2.08 (1.9)
CCFT Highest 3 Second Hold	25.74 (2)	28.96 (1.4)	25.62 (1.6)	27.49 (2)
DNFT Time Held (Seconds)	33.41 (22.7)	44.52 (21.9)	30.44 (18.2)	34.81 (16.8)
*Cervical Isometric Measurements*				
Flexion (Force)	31.01 (12.7)	44.11 (18.1)	44.33 (23.5)	42.88 (21.6)
Flexion (Percent)	19.77 (7.2)	28.16 (10.3)	27.52 (12.5)	27.29 (11.9)
Extension (Force)	42.89 (15.4)	52.52 (16.4)	51.85 (18.8)	49.98 (16.8)
Extension (Percent)	27.56 (9.1)	33.72 (8.8)	32.66 (9.4)	31.98 (9.1)
Left Side Flexion (Force)	26.96 (10.1)	35.05 (13.1)	33.56 (15.7)	32.94 (14.3)
Left Side Flexion (Percent)	17.29 (6)	22.52 (7.7)	21 (8.6)	20.94 (8)
Right Side Flexion (Force)	25.95 (10.3)	34.91 (12.3)	33.33 (14.8)	31.63 (14.1)
Right Side Flexion (Percent)	16.7 (6.3)	22.44 (7.4)	20.92 (8.1)	20.15 (8)
Left Rotation (Force)	20.66 (7.8)	28.17 (11.8)	25.94 (13.6)	26.06 (11.5)
Left Rotation Flexion (Percent)	13.22 (4.6)	18.14 (7.3)	16.45 (8.6)	16.58 (6.3)
Right Rotation (Force)	20.92 (8.3)	27.62 (11.1)	26.93 (14.5)	25.14 (11.2)
Right Rotation Flexion (Percent)	13.43 (5.1)	17.81 (7.2)	16.85 (8)	15.99 (6.2)
*Sway Scores – Balance*				
Feet Together	93.06 (10.7)	95.38 (7.7)	92.46 (8.3)	88.55 (17.1)
Tandem Right	86.66 (15.3)	87.81 (15.4)	83.02 (16)	79.15 (18.4)
Tandem Left	89.22 (11)	92.4 (8.6)	85.98 (16.7)	86.67 (14.1)
Single Leg Stance (R)	77.6 (19.1)	83.39 (16.4)	68.84 (24)	72.28 (20.2)
Single Leg Stance (L)	82.15 (14.9)	86.15 (12.8)	68.84 (23.1)	71.09 (20.2)
Overall Balance	85.74 (11.2)	89.03 (9.8)	79.83 (13.4)	79.55 (12.8)
*Sway Scores – Reaction Time*				
Simple	76.27 (8.1)	76.49 (8.1)	73.96 (7.9)	74.78 (6.9)
Impulse Control	62.33 (5.1)	64.61 (7.2)	62.62 (5.1)	63.88 (5.8)
Inspection	94.95 (4.7)	94.64 (4.1)	93.94 (5.7)	92.45 (5.1)

For the VIST exercises, there was a significant training effect for smooth pursuit, saccades, near-point convergence, peripheral vision, visual figure, joint position target, and cervical neuromotor control for the participants in the experimental group compared to the control (p values between <0.0001 and 0.005). For the clinical tests, there were significant improvements observed in the experimental group for cervical neuromotor control (CCFT3; p < 0.0001) and endurance (DNFT; p = 0.009). Additionally, each isometric strength measurement, balance stances, and total balance score on the Sway produced a significant improvement in the experimental group compared to the control at post-testing (p-values between <0.0001 and 0.005). For inspection time, there was a significant improvement for the experimental group (p = 0.008) but a significant difference was not attained for simple reaction time or impulse control pre-to post-in the experimental group compared to the control.

For the eye tracking data, across the total sample for all frequencies of smooth pursuit tested, a strong majority (n = 76–96) of the athletes were within the normal range of gain (0.85 ≤ gain≤1.0) at baseline. No significant changes were detected pre-to post-testing between the experimental and control for any of the measurements explored (slope, gain and phase) for any of the frequencies for smooth pursuits or saccades (latency, peak velocity, duration, or amplitude) in any direction (up, down, right, and left) (all group results presented in Tables 6 and 7).

**Table 6:** Smooth pursuit outcome characteristics, Mean (SD), of baseline to post-test.

Frequency	MC			UWA		
	Gain	Phase	Slope	Gain	Phase	Slope
Baseline						
1	0.9721 (0.067)	-3.3173 (4.414)	1.0057 (0.017)	0.9753 (0.067)	-3.2856 (4.134)	1.0108 (0.016)
2	0.944 (0.073)	-7.8899 (6.318)	1.0048 (0.019)	0.9463 (0.053)	-7.2104 (5.534)	1.0117 (0.015)
3	0.9064 (0.075)	-10.9944 (7.739)	1.0078 (0.019)	0.8899 (0.077)	-10.2985 (7.042)	1.0135 (0.013)
4	0.8716 (0.093)	-12.7208 (11.464)	1.009 (0.027)	0.8408 (0.092)	-11.8571 (10.864)	1.0145 (0.015)
Post-test						
1	0.9673 (0.045)	-5.0106 (3.674)	1.003 (0.019)	0.9555 (0.101)	-4.3445 (7.651)	1.0019 (0.022)
2	0.9545 (0.048)	-8.5276 (7.123)	1.0056 (0.017)	0.939 (0.084)	-8.0077 (5.581)	1.0039 (0.02)
3	0.9072 (0.092)	-12.5332 (9.454)	1.0068 (0.018)	0.9008 (0.079)	-11.1474 (9.813)	1.0029 (0.017)
4	0.8817 (0.083)	-15.1596 (10.092)	1.0083 (0.017)	0.871 (0.066)	-13.5062 (11.576)	1.0051 (0.02)

**Table 7:** Saccade outcome characteristics, Mean (SD), of baseline to post-test.

Direction	MC				UWA			
	Latency	Peak Velocity	Duration	Amplitude	Latency	Peak Velocity	Duration	Amplitude
Baseline								
Up	236.29 (34.4)	32.76 (6.2)	153.02 (24.7)	4.12 (0.5)	239.68 (51.2)	32.49 (8.1)	151.94 (26.7)	4.08 (0.5)
Down	253.14 (50.7)	35.34 (11.3)	151.61 (25.6)	4.24 (0.6)	245.02 (54.3)	34.07 (11.2)	153.25 (28.3)	4.15 (0.6)
Left	225.69 (44.4)	60.53 (8.2)	221.04 (29.2)	7.81 (0.4)	229.54 (63.4)	59.17 (7.4)	225.97 (29.8)	7.82 (0.5)
Right	224.28 (44.3)	59.2 (7.8)	224.16 (29.9)	7.81 (0.4)	229.89 (58.7)	58.79 (7.7)	228.99 (28.7)	7.84 (0.5)
Post-test								
Up	232.11 (36.3)	33.51 (6.6)	154.12 (25.8)	4.15 (0.5)	231.16 (44)	33.13 (6.4)	153.46 (23.8)	4.11 (0.5)
Down	251.43 (55.8)	34.35 (12.6)	153.41 (27)	4.2 (0.6)	245.82 (68.1)	34.68 (11)	149.8 (27.5)	4.19 (0.6)
Left	217.97 (36.2)	59.73 (10.6)	225.6 (27.8)	7.81 (0.4)	216.54 (46.2)	58.78 (7.4)	225.45 (30.1)	7.75 (0.4)
Right	222.15 (48.8)	59.04 (6.8)	223.15 (27.8)	7.79 (0.3)	222.71 (40.5)	58.27 (6.9)	226.41 (30.1)	7.74 (0.5)

In the exploration of whether there were changes in IVR scores in the experimental group after the period of time between cessation of training and the end of the season, a significant mean score improvement between post-test and follow-up was detected for near point convergence (p = 0.046) and a significant median score decrease between post-test and follow-up for smooth pursuit (p = 0.041) was detected. For the Clinical test outcomes, there was a significant increase in DNFT time (p = 0.0004) and decreases in isometric force measurements, with left side flexion and percent of body weight being the only measurements with significant decreases (p = 0.022 and 0.026 respectively). For the Sway tests, there were no significant changes between post–test and follow-up in the experimental group with exception of a significant decrease in simple reaction time (p = 0.008). The results are presented in Table 8.

**Table 8:** Outcome characteristics of post-test to follow-up scores in the experimental group.

	Post-test Mean (SD)	Follow-up Mean (SD)	P-Value
*IVR Exercise Scores*			
Smooth Pursuits†	234.45 (92.1)	214.15 (85.5)	0.041
Saccades	427.03 (68.1)	426.55 (65.7)	0.578
Near Point Convergence	184.08 (98)	203.03 (106.1)	0.046
Peripheral Vision†	342.14 (47.4)	336.74 (41.9)	0.268
Visual Figure†	409.93 (74.2)	403.03 (57.3)	0.229
Joint Position Target	237.63 (49.8)	231.41 (52.4)	0.366
Neuromotor Maze†	292.22 (72.1)	308.53 (65.3)	0.071
Cervical Posture†	314.63 (101.3)	308.89 (103.5)	0.93
Balance	253.07 (112.1)	254.52 (105.2)	0.903
*Clinical Test Outcomes*			
Convergence†	2.02 (2)	2.2 (1.9)	0.171
CCFT Highest 3 Second Hold†	28.96 (1.4)	28.76 (1.6)	0.577
DNFT Time Held (Seconds)	44.52 (21.9)	55.51 (28.3)	0.0004
*Cervical Isometric Measurements*			
Flexion (Force)†	44.11 (18.1)	41.38 (17.8)	0.461
Flexion (Percent)†	28.16 (10.3)	26.67 (10.5)	0.589
Extension (Force)†	52.52 (16.4)	50.34 (16.7)	0.206
Extension (Percent)†	33.72 (8.8)	32.79 (10.7)	0.28
Left Side Flexion (Force)†	35.05 (13.1)	32.76 (12.8)	0.022
Left Side Flexion (Percent)†	22.52 (7.7)	20.96 (7.6)	0.026
Right Side Flexion (Force)	34.91 (12.3)	32.6 (12.4)	0.064
Right Side Flexion (Percent)	22.44 (7.4)	20.86 (7.3)	0.068
Left Rotation (Force)†	28.17 (11.8)	26.34 (11.2)	0.091
Left Rotation Flexion (Percent)†	18.14 (7.3)	16.74 (6.5)	0.08
Right Rotation (Force)†	27.62 (11.1)	26.67 (12.4)	0.337
Right Rotation Flexion (Percent)	17.81 (7.2)	17.01 (7.6)	0.544
*Sway Scores – Balance*			
Feet Together†	95.38 (7.7)	94.94 (8)	0.292
Tandem Right†	87.81 (15.4)	89.36 (11.7)	0.604
Tandem Left†	92.4 (8.6)	92.71 (8)	0.272
Single Leg Stance (R)†	83.39 (16.4)	83.8 (14.9)	0.899
Single Leg Stance (L)†	86.15 (12.8)	86.29 (14.4)	0.984
Overall Balance†	89.03 (9.8)	89.42 (8.5)	0.833
Sway Scores – Reaction Time			
Simple	76.49 (8.1)	73.77 (8.5)	0.008
Impulse Control	64.61 (7.2)	63.11 (6.6)	0.084
Inspection†	94.64 (4.1)	94.54 (5.5)	0.962

† Wilcoxon signed-rank test; Neuromotor maze (n = 50) post-test and (n = 30) follow-up; remainder of IVR scores included (n = 71–73) at post-test and (n = 62–66) at follow-up. IVR = Virtual Reality; CCFT = Cranial-Cervical Flexion Test; DNFT = Deep-Neck Flexor Test. R = right; L = left. All cervical isometrics measured in pounds of force; percentage should be interpreted as percent of max force calculated based off total body weight. All balance stances were completed with the participants eyes closed.

Comparing if there were differences in performance between the experimental and the control groups after a period of non-training in IVR, the regression analysis of change scores revealed that the experimental group demonstrated significantly better performance in saccades (p = 0.011) and visual figure (p = 0.002) compared to the control group. For the clinical test outcomes, only the DNFT demonstrated significant improvement in the experimental (p = 0.009). On the Sway tests, the experimental group consistently outperformed the control, with tandem right (p = 0.002) and single leg stance left (p = 0.033) and overall balance (p = 0.024) demonstrating significance. Conversely, the control group demonstrated significantly better performance on the near-point convergence IVR exercise than the experimental (p = 0.042) and simple reaction time on the Sway was also significantly faster after the period of non-training in the control (p = 0.036). The regression results are presented in Table 9.

**Table 9:** Regression analysis of score changes comparing experimental to control during period of non-training.

Tests	Coefficient Estimates (Std Err)	95% CI	P-value
*IVR Scores*			
Smooth Pursuits	-1.03 (15.1)	(-30.92, 28.85)	0.946
Saccades	44.45 (17.2)	(10.41, 78.5)	0.011
Near Point Convergence	-30.52 (14.8)	(-59.93, -1.11)	0.042
Peripheral Vision	11.95 (8.7)	(-5.38, 29.28)	0.175
Visual Figure	48.51 (15.3)	(18.16, 78.87)	0.002
Joint Position Target	10.29 (13.7)	(-16.77, 37.35)	0.453
Neuromotor Maze	26.62 (13.8)	(-1, 54.24)	0.059
Cervical Posture	5.05 (19)	(-32.64, 42.75)	0.791
Balance	-11.26 (18.9)	(-48.79, 26.26)	0.553
*Clinical Test Outcomes*			
Convergence	-0.09 (0.2)	(-0.47, 0.29)	0.627
CCFT Highest 3 Second Hold‡	-0.46 (0.3)	(-0.99, 0.06)	0.084
DNFT Time Held (Seconds)	10.86 (4.1)	(2.83, 18.9)	0.009
*Cervical Isometric Measurements*			
Flexion (Force)	-0.71 (2.2)	(5.03, 3.6)	0.744
Flexion (Percent)	-0.64 (1.3)	(-3.3, 2.02)	0.635
Extension (Force)	-0.06 (2)	(-3.97, 3.84)	0.975
Extension (Percent)	0.26 (1.3)	(-2.34, 2.86)	0.842
Left Side Flexion (Force)	-1.49 (1.4)	(-4.33, 1.34)	0.3
Left Side Flexion (Percent)	-1.13 (0.9)	(-2.89, 0.62)	0.203
Right Side Flexion (Force)	-0.2 (1.4)	(-3.07, 2.66)	0.889
Right Side Flexion (Percent)	-0.29 (0.9)	(-2.12, 1.54)	0.755
Left Rotation (Force)	-0.98 (1.3)	(-3.63, 1.66)	0.462
Left Rotation Flexion (Percent)	-0.58 (0.8)	(-2.26, 1.1)	0.494
Right Rotation (Force)	1.12 (1.4)	(-1.61, 3.85)	0.419
Right Rotation Flexion (Percent)	0.5 (0.9)	(-1.19, 2.2)	0.557
*Sway Scores – Balance*			
Feet Together	3.73 (2.3)	(-0.88, 8.34)	0.112
Tandem Right	8.77 (2.7)	(3.39, 14.16)	0.002
Tandem Left	2.64 (1.8)	(-0.97, 6.26)	0.151
Single Leg Right	5.27 (3)	(-0.67, 11.21)	0.082
Single Leg Left	7.01 (3.2)	(0.58, 13.45)	0.033
Overall Balance	3.8 (1.7)	(0.5, 7.09)	0.024
*Sway Scores – Reaction Time*			
Simple	-2.62 (1.2)	(-5.07, -0.18)	0.036
Impulse Control	-1.95 (1.1)	(-4.08, 0.18)	0.072
Inspection	1.71 (0.9)	(-0.03, 3.46)	0.055

‡ Ordinal logistic regression; IVR = Virtual Reality; CCFT = Cranial-Cervical Flexion Test; DNFT = Deep-Neck Flexor Test: all cervical isometrics measured in pounds of force; percentage should be interpreted as percent of max force calculated based off total body weight. All balance stances were completed with the participants eyes closed.

**Figure 2:**
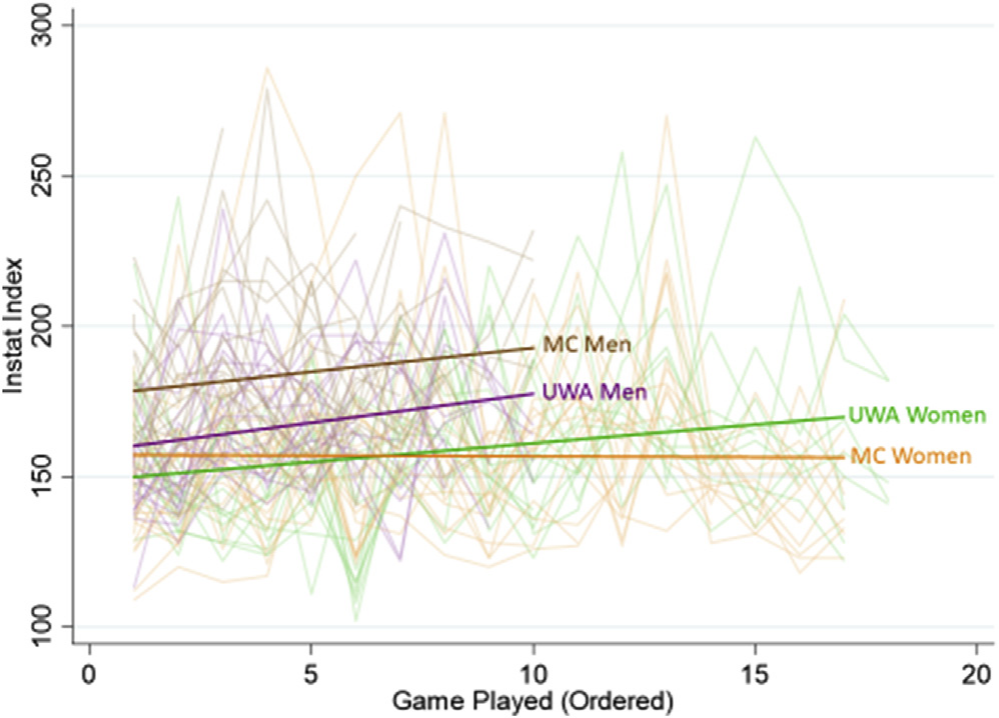
Trajectory of InStat Index Scores for each team across the 2019 soccer season.

Regarding the far-effects, there was no difference in the reported injury incidence rate across the 2019 soccer season between the experimental and control groups (9.9 per 1000 AEs at UWA and 15.2 per 1000 AEs at MC; p = 0.315). Further, higher IVR exercise scores measured at pre- or post-test 1 did not confer a significant protective effect on injury incidence. For on-field game performance, the InStat Indices were widely variable across all players on all teams across the season. Figure 2 displays individual player index scores for each game and the overall mean score for each team across the season. Across the soccer season, 22 men and 22 women at MC and 21 men and 16 women at UWA received an InStat score in one or more competitions, contributing to these outcomes. MC men consistently outperformed UWA men, both teams proportionally increasing on-field performance across the season. The MC women demonstrated consistent, nearly unchanged mean performance scores across the season, while the UWA women mean performance scores rose. Exploring the association between IVR exercise performance and on-field performance within the entire cohort of athletes, it was found that a higher score on smooth pursuits was associated with a higher InStat Index score (p = 0.022), specifically, one additional point on the InStat Index score per 25 points scored in smooth pursuit. No other IVR exercise scores were significantly associated with on-field performance as measured through this index.

## 4 Discussion

The purpose of this investigation was to determine the effects of a virtual immersive sensorimotor training intervention utilizing activities delivered primarily through IVR. Training effects, near-effects, and far-effects were quantified and compared between an experimental and control group and associations between IVR exercise performance and far-effects were explored.

With reference to the principles of neuroplasticity proposed by Kleim and Jones [35], the VIST exercises trained specific components of sensorimotor control, by utilizing sensory stimuli to generate a precise action goal. The exercises were then repeated across time as they progressed in difficulty and complexity to improve the efficiency of the sensorimotor control system. The results indicate significant training effects in a majority of the IVR exercises for the experimental group. The training effects observed are not unexpected, given that the testing environment was exactly the same as the training environment.

To further investigate whether the VIST exercises were successfully training what was being targeted and that the measurements obtained by the IVR sensors were measuring changes in specific sensorimotor abilities, the near-effects provide additional insight. Here, the results demonstrate significant improvements in all measures of cervical neuromotor control, endurance, and isometric strength, standing balance, and inspection time. This is specifically helpful in concluding that although the IVR exercises called “cervical posture” and “balance” did not produce identifiable training effects, it is likely that they contributed to improvement in these abilities. In this case, the sensor measurement through the IVR headset and/or software algorithm calculation of the performance score may not reflect actual performance. All together, the significant improvements in the clinical tests provide evidence of a transfer effect for the training of these complex skills. Previous research has demonstrated that sensorimotor control is a trainable human ability, even in athletes with unimpaired sensorimotor control [5, 36, 37]. In similar fashion, our results demonstrate that IVR provided an effective training environment for exercises aimed at enhancing sensorimotor control.

To our knowledge, research evidence exploring the use of a 3-dimensional IVR training environment with exercises specifically designed to enhance the various neurologic processes which contribute to overall sensorimotor control, including vestibular, visual and oculomotor activities, cervical neuromotor control training, coordination, and postural/balance is non-existent. Other research describing transfer of complex sensory-motor skills from a virtual to a real-world environment has demonstrated mixed results and usually involves an upper-extremity task [38]. One proposed reason that many motor skills may not successfully transfer out of a virtual to a real-world environment is because the sensory information available to the person during the training in a virtual world does not match the sensory information required for performance in the real-world [38]. One of the reasons that a positive transfer may have been easier to achieve in this present study is that the effects of gravity, postural sway, and head movement were used by the participants during the training and feedback associated with their use was included as part of the IVR training environment. The headset sensors, six degrees of freedom capability (which provides accurate positional information about the virtual environment relative to movement of the user), and score derivation enabled accurate knowledge of performance and knowledge of results to further drive motor learning [35].

Conversely, the IVR exercises aimed at oculomotor control demonstrated consistent improvement in IVR performance, but the near-effect outcomes did not produce significant findings. It is believed that this is likely a result of two compounding factors. First, we were working with healthy athletes with non-impaired eye movements, trying to improve upon “normal”, and second, the eye tracker was unable to detect change in these eye movement parameters. Unfortunately, a non-research grade eye tracker was used to measure oculomotor control with a frame rate of 90 Hz and a 5% zone of error in detection of precise eye position relative to the position of the object on the screen. The tracker simply lacked the frame rate and the precision of visual location on the computer screen necessary to enable us to perceive minute changes in oculomotor control. This lack of transfer may also be explained by the fact that the training was performed in a 3-dimensional environment whereas the testing was completed on a 2-dimensional screen, making the sensory cues different in the transfer test than in training [38]. Future research will be conducted with a research grade eye-tracker to explore if there is a training effect on oculomotor control while completing tasks in the 3-dimentional virtual environment.

One positive indicator of overall visual-perceptual eye function is the improvement in inspection time. Inspection time is more indicative of functional visual-cognitive performance and serves as a measurement of complex reaction time [39]. This test required the participant to determine which line, presented on the left or right side of the screen was longer. This requires visual inspection, perception, cognitive selection, and response through a movement of the device (right or left). Related research findings have demonstrated the importance of visual search strategies, speed of visual fixations, and perceptual-cognitive processes for soccer performance [40]. It is believed that the significant improvement in inspection time found in this present study was produced through the composite training effect from the exercises for visual perception and oculomotor control.

Another interesting finding related to the training effect is that, after a period of no training in the experimental group, a significant degradation was only evident for one of the IVR exercises (smooth pursuit). This finding is interesting because smooth pursuit performance in IVR was the only IVR exercise that was significantly associated with on-field performance. It should be noted that this IVR exercise required the participants to complete smooth pursuit eye movements, in response to an object moving in the 3-dimensional visual field, while coordinating the movement of their head/neck and activating a manual controller. An exercise such as this may encourage the use and development of visual exploratory activity (VEA) during the performance of soccer, which is an important ability for on-field success [9]. This may be an exercise to consider continuation of training across an athletic season to boost performance.

Looking at far-effects, on-field performance and rate of injury did not produce significant differences between groups. While this is a major goal for this line of work, attaining significant findings in these areas is a lofty stretch for several reasons. First, with regards to the injury rate, the sample size required to accumulate the number of exposures and incident cases to detect an effect to a population level intervention is large, much larger than the sample utilized in this pilot study. Second, each team had a different athletic trainer and a different coach. Each team, coach, and trainer have different norms and practices relative to injury. This provides variability that cannot be accounted for in analysis. Research describing sensorimotor types of training (e.g. oculomotor, balance, and general neuromuscular training) in athletes have demonstrated significant injury risk reductions post-training [6, 36, 37]. A future study, powered with a large enough sample, is needed to quantify whether this intervention imparts a protective effect against sports-injury.

With respect to on-field performance, the InStat Index was used because it provides a metric of game performance which is recognized by many university and professional teams. There are, however, several factors limiting the scope of what can be inferred by this index. First, the InStat Index measures performance outcomes, but does not provide any insight into the cognitive/visual search strategies and other neuromotor processes that contribute to on-field decision making and production of skilled movement, both of which are important functional abilities related to this intervention. Second, game performance is heavily influenced by a myriad of factors, which are difficult to measure and quantify, including the field conditions, weather, team dynamics, level of stress, opposing team playing tactics, coaching decisions, and others. It is not known how much the IVR training verses these other factors contributed to any specific game performances or match outcomes.

Perhaps a more compelling source of measurement of game performance for coaches and athletes is wins and losses, which is also heavily influenced by all the factors discussed above. The institutions participating in this research study were from the same conference, played each other, and many similar opponents. Therefore, relative performance as a team within the conference provides ecological evidence related to this work. In 2019, the men and women's teams at MC (experimental group) were regular season conference champions. The women finished with a perfect conference record of 12 wins and 0 losses and the men finished with a conference record of 7 wins, 0 losses and 3 ties, a 0.85 winning record. Prior to 2019, the MC soccer teams had not attained this feat within their conference simultaneously. As a point of discussion for this study, this is an interesting and unexpected outcome, but whether there is any association between the IVR training provided and the overall success of the MC teams across the season of play is unknown.

In addition to those presented above, additional limitations of this project include the inability to blind participants or the outcome assessors to the allocation of participants. However, because many of the outcomes were measured electronically with little to no assessor input (including IVR scores, balance, reaction time, isometric strength, eye tracking, and on-field performance), we believe that observer expectation bias was minimized. It is, however, possible that participant expectation was enhanced in the experimental group and this potential impact is not known. Further, the differences between the institutions in the use of strength and conditioning, specifically neck strengthening exercises, likely contributed to the improvements in cervical outcomes in the experimental group, and these effects cannot be separated from the effects from the IVR exercises alone. Similarly, differences in other aspects of training, primarily on-field practice and soccer drills, is not known and was variable across each team and may or may not have influenced the findings reported here. It should also be noted that 63% of the athletes in the experimental group participated in a research study in 2018, completing sensorimotor training activities (in and out of IVR) [5]. In the event of any carryover, statistical methods were utilized to account for differences in baseline score performance. It is possible that these athletes’ sensorimotor systems were “primed” in a manner to respond more efficiently to specific training, contributing to enhanced outcomes, but this is not known. Last, the determination of dosage for IVR training was based on related work but it is possible that this was not sufficient to produce the optimal level of training, to achieve the scope of improvement possible, and to a level that could have produced on-field performance and injury rate improvements.

In conclusion, this research study provides evidence supporting the use of novel VIST exercises to improve measures of sensorimotor control in healthy soccer athletes. There is evidence to support this training to improve clinical outcome measures of sensorimotor and cervical neuromotor control. Although far-effects of injury incidence and on-field performance were not demonstrated in this present study, it is well agreed upon that optimal sensorimotor control is important for athlete safety in contact sports. Additional research powered with a sufficient sample-size, across more than one season of play, will be required to quantify whether there is a meaningful impact of this type of training and enhancement on risk of injury and performance.

## Declarations

### Author contribution statement

J. Reneker: Conceived and designed the experiments; Performed the experiments; Analyzed and interpreted the data; Contributed reagents, materials, analysis tools or data; Wrote the paper.

M. Reneker: Conceived and designed the experiments; Wrote the paper.

W. Pannell and R. Babl: Conceived and designed the experiments; Performed the experiments; Wrote the paper.

F. Adah: Performed the experiments; Wrote the paper.

Y. Zhang and S. Lirette: Analyzed and interpreted the data; Wrote the paper.

### Funding statement

This work was supported by the Federal Office of Rural Health Policy (FORHP), Health Resources and Services Administration (HRSA), U.S. Department of Health and Human Services (HHS) (6 U66RH31459-02-03).

### Competing interest statement

The authors declare the following conflict of interests: J. Reneker has Receipt of Intellectual Property Rights/Patent Holder: Patent 21764.8 (pending) entitled: VIRTUAL IMMERSIVE SENSORIMOTOR TRAINING SYSTEM FOR IMPROVING FUNCTIONAL PERFORMANCE.

### Additional information

No additional information is available for this paper.
